# Coral mucus as a reservoir of bacteriophages targeting *Vibrio* pathogens

**DOI:** 10.1093/ismejo/wrae017

**Published:** 2024-01-31

**Authors:** Esther Rubio-Portillo, Sophia Robertson, Josefa Antón

**Affiliations:** Department of Physiology, Genetics and Microbiology, University of Alicante, Alicante 03690, Spain; Department of Physiology, Genetics and Microbiology, University of Alicante, Alicante 03690, Spain; Department of Biological Sciences, Northern Arizona University, Flagstaff, AZ 86011, United States; Department of Physiology, Genetics and Microbiology, University of Alicante, Alicante 03690, Spain; Multidisciplinary Institute of Environmental Studies Ramon Margalef, Alicante 03690, Spain

**Keywords:** vibriophage, coral mucus layer, Vibrio mediterranei

## Abstract

The increasing trend in sea surface temperature promotes the spread of *Vibrio* species, which are known to cause diseases in a wide range of marine organisms. Among these pathogens, *Vibrio mediterranei* has emerged as a significant threat, leading to bleaching in the coral species *Oculina patagonica*. Bacteriophages, or phages, are viruses that infect bacteria, thereby regulating microbial communities and playing a crucial role in the coral’s defense against pathogens. However, our understanding of phages that infect *V. mediterranei* is limited. In this study, we identified two phage species capable of infecting *V. mediterranei* by utilizing a combination of cultivation and metagenomic approaches. These phages are low-abundance specialists within the coral mucus layer that exhibit rapid proliferation in the presence of their hosts, suggesting a potential role in coral defense. Additionally, one of these phages possesses a conserved domain of a leucine-rich repeat protein, similar to those harbored in the coral genome, that plays a key role in pathogen recognition, hinting at potential coral–phage coevolution. Furthermore, our research suggests that lytic *Vibrio* infections could trigger prophage induction, which may disseminate genetic elements, including virulence factors, in the coral mucus layer. Overall, our findings underscore the importance of historical coral–phage interactions as a form of coral immunity against invasive *Vibrio* pathogens.

## Introduction

Forty coral diseases have thus far been described, although only a few coral pathogens, including six *Vibrio* species, have been identified so far [1]. The dynamics of coral diseases are influenced by shifting interactions in the coral holobiont, a meta-organism formed by a dynamic multipartite relationship between the cnidarian host and its microbiota. Environmental factors, particularly elevated seawater temperature, contribute to alterations in the coral microbiome, resulting in dysbiosis within the commensal microbial community. This dysbiosis, in turn, creates favorable conditions for the proliferation of opportunistic pathogens such as *Vibrio* species that can act as secondary pathogens [2]. In addition to the diverse cellular microbes, coral microbiomes harbor a highly diverse assemblage of viruses [3-5] that could be involved in the modulation of coral health. Indeed, it has been observed that the abundance of virus-like particles increases during stressful conditions [6] or bleaching events [7].

In the coral holobiont, higher abundances of viruses occur within the surface mucus layer (SML), where a diverse range of viruses have been described, encompassing bacterial, archaeal and eukaryotic viruses [5]. The abundance and diversity of phages in the SML strongly suggests that they play an important role in regulating the abundances of specific bacterial populations through targeted infection and lysis [8]. This process that allows phages to exert control over microbial community populations has been proposed to form a “lytic barrier” against bacterial pathogen colonization, suggesting that viruses should be classed as an active part of the coral nonhost-derived immunity [8, 9]. According to this hypothesis, phages potentially protect the coral host from fast-growing and highly abundant bacterial populations such as vibrios.

It is well established that phage-mediated modifications can lead to significant changes in *Vibrio* phenotypes, impacting the fitness and virulence of the hosts (reviewed in Molina-Quiroz *et al*. [10]). Therefore, expanding our knowledge about *Vibrio* phages present in SML is of great importance not only from an ecological point of view but also for the potential use of these phages for prophylaxis and therapies to prevent or treat coral diseases [11]. Thus, the isolation and characterization of phages that infect coral pathogens is essential to increase the collection of potential candidates for therapeutic assays.

The isolation and characterization of phages against human pathogenic *Vibrio* species have a long-standing history (for review, see Plaza *et al*. [12]). Regarding *Vibrio* coral pathogens, most studies have primarily focused on isolating and characterizing phages that specifically target *Vibrio coralliilyticus* [13-17]. This particular focus may be attributed to the worldwide distribution of this bacterium and its economic significance due to its ability to infect shellfish. Another well-known coral pathogen is *V. mediterranei* [18], an emerging pathogen in the Mediterranean Sea, responsible not only for coral diseases but also for mortalities in scallops or the giant fan mussel *Pinna nobilis* [19]. Given the warming rates in the Mediterranean Sea that exceed the global average [20], the impact of this emerging pathogen on Mediterranean fauna is expected to be significantly amplified in the next few decades. However, to the best of our knowledge, there have been only two studies conducted on *V. mediterranei* phages and only one phage isolated, which has shown high efficacy in treating diseases in seaweeds [21, 22].

In this study, we combined phage isolation with a metagenomic approach to characterize the viral community associated with the SLM of the coral *Oculina patagonica*, as well as to investigate changes in this community under heat stress. This coral has been considered an excellent model system for coral bleaching studies in the Mediterranean Sea, and it is well established that *V. mediterranei* is part of its microbial community and can act as secondary pathogen under heat stress. Thus, the study of the system may shed light on the tri-partite interaction between corals, their vibrio pathogens, and the vibriophages controlling them. In this work, to enhance phage isolation, we conducted host-targeted phage enrichments and successfully isolated 10 phages, which belonged to two different species within the same genus. Our metagenomic results suggest that these phages are typically low-abundance specialists within the coral mucus layer but with a high infectivity against *Vibrio* strains previously present in the system, which suggests that these phages may serve as an acquired immune system for corals to defend against pathogens. Furthermore, during the lytic infection the induction of one *V. mediterranei* prophage was detected; this phage was also identified and described and its genome sequenced. Consequently, this study not only is an investigation into viral diversity in *O. patagonica* but also represents a significant step forward in understanding vibriophages as a form of coral defense system against invasive *Vibrio* pathogens.

## Material and methods

### Sample collection and processing

**Figure 1:**
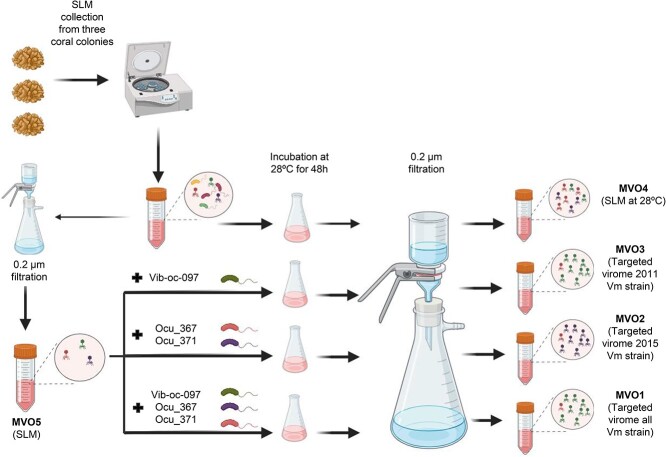
Schematic representation of the viromes utilized in the present study; this figure was created using BioRender (https://biorender.com/).

The *O. patagonica* samples were collected in June 2019 from the marine protected area of Tabarca in Spain (38°09′59´´N, 0°28′56´´W). Three coral samples were transported to the laboratory in a cooler within the next 2 h and immediately processed to obtain the secreted mucus [18]. Five milliliters of the mucus obtained from the three coral colonies was used to extract the deoxyribonucleic acid (DNA) and to study the natural viral assemblage (hereafter sample MVO5), the supernatant was filtered through 0.2-μm polyethersulfone (PES) filters to eliminate cells and obtain the coral viral fraction. Then, the sample was concentrated to ~200 μl using the Amicon Ultra-15 Centrifugal Filter Devices 100 K (Merck Millipore, Germany. In addition, 5 ml of the coral mucus were incubated at 28°C during 48 h to assess the effect of the increase of seawater temperature on the natural phage community (MVO4, which was also referred to as “stressed” virome) (Fig. 1).

### Targeted viromes

With our previous unsuccessful attempts to isolate *V. mediterranei* phages using seawater as the phage source, in this study, a total of three phage enrichment cultures, referred to as “targeted” viromes, were created using aliquots of the coral mucus sample as phage source. The coral mucus sample (250 μl) was used to inoculate 10 ml of Luria-Bertani (LB) broth plus 3% NaCl (LBNaCl) together with 100 μl of the corresponding vibrio host(s) and incubated at 28°C with 180 rpm shaking for 48 h. Three strains of *V. mediterranei* were used as hosts for targeted viromes: strain Vib-oc-097 (CECT 30098), a causative agent of coral bleaching originally isolated in 2011 from a bleached colony of *O. patagonica* [18], together with two additional strains (OcU_367 and OcU_371) isolated also from *O. patagonica* during the marine heatwave registered in 2015 [23] (Supplementary Table 1). A total of three targeted viromes were created by incubating coral mucus with the following hosts: (i) the three *V. mediterranei* strains together (MVO1), (ii) the two *V. mediterranei* strains isolated in 2015 (MVO2), and (iii) the *V. mediterranei* strain isolated in 2011 (MVO3). After the incubation, samples were centrifuged and filtered to eliminate cells, as was described above. Two milliliters of filtrate were kept at 4°C until used for phage isolation, and the rest was concentrated using Amicon Ultra-15 Centrifugal Filter Devices 100 K (Merck Millipore, Darmstadt, Germany) for DNA extraction.

### *V. mediterranei* phage isolation, purification, and propagation

The *V. mediterranei* strains were used as hosts for isolating phages from all virome samples (MVO1, MVO2, MVO3, MVO4, and MVO3). Initially, spot testing was used to isolate the phages. The host bacterial lawn was made by using LBNaCl plus 0.7% agar and overlaying host bacterial suspensions on top of LBNaCl plates (2% agar). When the agar overlays were solidified, viral concentrates from the targeted virome incubations were spotted (10 μl) onto the lawns and the plates were then incubated at 30°C for 24 h. The agar spot with lysed cells was cut and resuspended into sterile seawater, centrifuged, and then filtered again. The supernatants were used to isolate and purify individual phages from spot filtrates with a standard plaque assay, using a series of 10-fold dilutions. Exponential phase host cultures were mixed with dilutions of spot filtrates and incubated at room temperature for 30 min for phage adsorption. These suspensions were mixed with the LBNaCl top-agar and poured onto LBNaCl agar plates. To purify the phages, isolated plaques were picked with a pipette and suspended in sterile seawater. The suspensions were centrifuged and the supernatants were filtered through 0.2-μm PES filters. This process of purifying individual plaques through a plaque assay was repeated at least three times to ensure purity of the isolated phages.

### Host range

A collection of 19 *V. mediterranei* and *V. coralliilyticus* strains isolated from different marine invertebrates was used to determine the host range of each isolated phage (Supplementary Table 1). *V. coralliilyticus* was included in order to determine the ability of these viruses to infect other *Vibrio* species. The susceptible hosts were first detected with a spot test using direct, 10- and 100-fold dilutions. The susceptible hosts were then exposed to the same phage titer [1 × 10^11^ virus-like particles (VLPs)/ml], and infectivity was quantified by plaque assay and counting plaque-forming units (PFUs).

### Transmission electron microscopy, pulsed-field gel electrophoresis, and virus deoxyribonucleic acid extraction

One milliliter from each viral culture was ultracentrifuged at 50 000 × g (1 h 20°C) and processed for transmission electron microscopy as described previously [24]. VLPs were observed in a Jeol JEM-2010 transmission electron microscope (JEOL Manufacturer, Tokyo, Japan) operating at 200 kV.

Fifty microliters of the ultracentrifuged sample (~5 × 10^8^ virus-like particles) were used to determine the viral genome sizes by pulsed field gel electrophoresis (PFGE) as described previously [25].

Concentrated viromes as well as isolate phages were used for DNA extraction using a QIAamp UltraSens virus kit (Qiagen). Sequencing of viral DNA was performed using the Nextera XT DNA Library Preparation Kit and the MiSeq System (Illumina) (at FISABIO, Valencia, Spain).

### Deoxyribonucleic acid sequence analyses

The presence of bacterial contamination in the virome samples was assessed by determining the proportion of bacterial 16S ribosomal ribonucleic acid (rRNA) genes and single-copy bacterial genes using ViromeQC [26], and the Nonpareil tool [27] was used to estimate the coverage of the community in each metagenome dataset. The sequence reads from viromes were quality trimmed by Trimmomatic [28] and then assembled into contigs with metaSPAdes [29]. Viral contigs larger than 5 kb were extracted from all assembled viromes, and the viral genomes were further validated using VIBRANT [30]. The completeness of each viral genome was estimated using the CheckV v0.6.0 pipeline [31], which was also employed to identify viral contigs containing provirus integration sites or integrase genes, allowing inference of putative temperate lifestyles. To group viral sequences into viral operational taxonomic units (vOTUs), cdhit was used with a 95% average nucleotide identity and 80% alignment fraction threshold for the smallest contig [32]. Representative genomes for each vOTU were then annotated using Pharokka [33]. IPHoP, which integrates all currently available virus host prediction methods and builds a machine learning framework, was used to predict virus–host interactions [34].

To calculate the relative abundance of each vOTU within the viral community, a read-mapping approach with BBmap was employed [35]. This was followed by estimating the central 80% of the truncated average sequencing depth (TAD80) for each genome [27]. A genome was considered present in a sample when at least 75% of the viral genome recruited reads at 95% of identity.

Isolated phage genomes were processed as was described above for the viromes. Whole viral genome comparisons (in nucleotides) were performed by Viridic [36] and genome alignments were drawn by Easyfig platform. A viral proteomic tree including published viral genomes was inferred using ViPTree [37]. The largest and most complete genome of each cluster was selected as “group reference genome” and annotated using virClust [38]. Moreover, the software strategy, a powerful and fast sequence homology detection method, was used to predict functions of unknown proteins detected in the phage genomes [39].

### Phage detection in natural coral samples by polymerase chain reaction amplification

Two primer pairs were manually designed to amplify each of two identified phage species, based on the sequence alignment of the 10 phages. Thus, the following primer sets were used for *Planavibriovirus adelos* and *P. lipares*, respectively: PadelosF (GATAGGTATTGCGCGGTTAGA) and P-adelosR (CATTTGACACTCGGCGATAAAG), and PliparesF (AATGTGGGACATAGTGCAAT) and PliparesR (CTGTCTGATGTCGCTCATAA). PCR reactions were optimized using the corresponding viral DNAs as templates at varying annealing temperatures. When the reaction was performed with an annealing temperature of 55°C, a single band was consistently detected via electrophoresis, and the specific product for each primer set was checked by sequencing. Subsequently, these primers were employed for the detection of these phage species in environmental samples. DNA extracted from coral colonies collected during a 2015 heatwave, during which *V. mediterranei* was identified through culture-dependent techniques [23], was used as template.

## Results and discussion

### Viral community associated with the *O. patagonica* mucus layer

Five viromes from the *O. patagonica* mucus layer were analyzed (Fig. 1). The VIROME QC analysis revealed that bacterial contamination was negligible (see Table 1) and Nonpareil analysis indicated that sequence coverage values exceeded the recommended 60% threshold for obtaining meaningful, unbiased biological conclusions from metagenomes [26]. A total of 70 different viral operational taxonomic units (vOTUs) were identified, comprising complete or high-quality (*n* = 33) and medium-quality (*n* = 37) vOTUs. About 50% of these vOTUs exhibited similarity to reference genomes and were classified as dsDNA phages belonging to the class *Caudoviricetes*. This finding agrees with previous analyses, which utilized diverse coral, prokaryotic, and viral metagenome and meta-transcriptome datasets, revealing the prevalence of dsDNA viruses belonging to the *Caudovirales* order within the coral holobiont [5].

**Table 1:** Characteristic of the viral metagenomes (metaviromes) sequenced in this work.

Samples	Nonpareil		ViromeQC		Metavirome size (Mbp)	GC (%)	Contigs	Contigs >5 kb	Max contig length (bp)	Viral OTUs	*Vibrio* phages
	Coverage	Diversity	16S rRNA (%)	Bacterial marker genes (%)							
MVO1 (all Vm strains)	98.91	10.44	0.001	0.005	259.10	56.35	688	6	44 307	12	7
MVO2 (OcU_367 and OcU_317)	99.75	10.77	0.005	0.000	581.00	57.52	64	1	44 825	9	5
MVO3 (Vic-Oc-097)	98.95	10.75	0.001	0.003	471.10	56.12	1598	9	48 550	12	7
MVO4 (coral mucus at 28°C)	94.21	10.98	0.016	0.009	511.90	44.26	19 107	49	48 550	16	7
MVO5 (coral mucus)	93.19	11.66	0.007	0.015	3832.76	55.11	165 273	553	317 471	54	0

**Figure 2:**
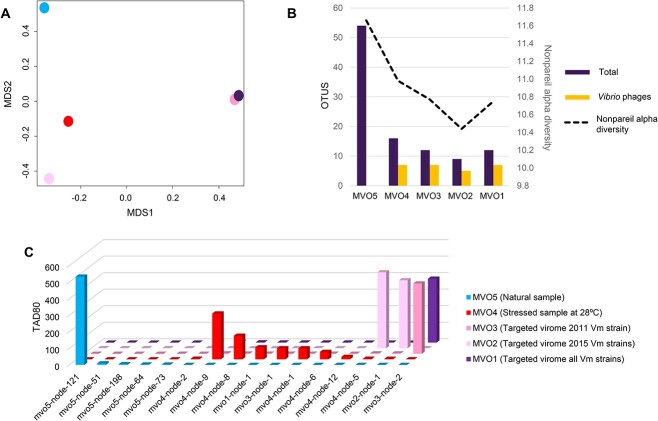
Characterization of coral mucus viromes; (A) two-dimensional NMDS plot based on the MASH distance; (B) bar graph showing the number of total viral OTUs detected and number of vibriophages detected; the dotted line shows the Nonpareil diversity in each virome; (C) bar plot showing read depth data truncated to the middle 80% (TAD80) of depth values for the most abundant viral OTUs identified in each virome.

The viral community naturally associated with *O. patagonica* mucus was compared to the viral communities generated after incubation with *V. mediterranei* strains (targeted viromes MVO1, MVO2, and MVO3), as well as the viral community selected after mucus incubation at 28°C (stressed virome, MVO4). MASH distances showed that the natural and the stressed viral communities were different, which suggests that the incubation temperature, 2 degrees above the average seawater temperature, selected some bacterial hosts and their phages. The distances between the natural sample and each of the targeted viromes were higher than the distance between the natural and stressed sample; among the targeted virome comparisons, MVO1 (consisting of the three *V. mediterranei* strains together) and MVO3 (the *V. mediterranei* strain isolated in 2011) showed the highest similarities to the natural sample (Fig. 2A). Nonpareil analyses revealed a decrease in diversity within all the targeted viromes and the stressed virome when compared to the natural virome (Fig. 2B, Table 1). This decrease was accompanied by a decrease in the number of recovered phage genomes. In the targeted virome with hosts from 2015 (MVO2), only one potential phage genome was assembled, indicating a clear enrichment of this phage in the viral community (Table 1). Furthermore, a decrease in detected vOTUs was also observed in the stressed virome, though the number of vOTUs was still higher than in the targeted viromes (Fig. 2B).

*In silico* prediction of bacterial hosts for the viral genomes revealed that the number of phages infecting *Vibrio* spp. was higher not only in the targeted viromes but also in the stressed virome (Fig. 2B; Supplementary Table 2), whereas these phages were not detected in the natural sample. The enrichment in the targeted viromes is expected given that mucus samples were incubated with *Vibrio* hosts. In the stressed virome, high temperatures could be favoring vibrio growth and the consequent selection of vibriophages. In fact, *Vibrio* genus abundance in SLM of the coral *O. patagonica* tends to increase during stressful conditions [23, 40].

Most of the assembled phages in the natural coral virome were not detected in the targeted or stressed viromes (Fig. 2C). Conversely, viral contigs enriched in the targeted viromes were below the detection level in the natural sample (Fig. 2C) but were present in the stressed sample incubated at 28°C and corresponded to phages able to infect different *V. mediterranei* strains, as unveiled by phage isolation (see below). Differences were also detected in virus-encoded auxiliary metabolic genes (vAMGs) detected in the natural samples that were not present in the other viromes. This was the case of the queuosine biosynthesis genes *queC*, *queD*, and *queE*, along with the queuine transfer RNA (tRNA)-ribosyltransferase, which were the most common AMGs in MV05. Genes from the queuosine biosynthesis pathways have been previously described in marine phages that seem to use it to modify their DNA to escape host restriction endonucleases or to increase translation efficiency by redirecting global host protein synthesis through queuosine-containing tRNAs [41, 42].

### Virus isolation

Each of the three *V. mediterranei* strains used as hosts in the targeted viromes was challenged with each of the five viromes (MVO1 to MVO5), and only some combinations yielded plaques on host lawns and were thus used for subsequent virus isolation (Supplementary Table 3). More specifically, plaques were obtained from targeted viromes (MVO1 to MVO3) whereas no plaques were obtained using either the natural mucus (MVO5) or the stressed sample (MVO4) as viral sources. Phages able to infect strains isolated in 2015 (Ocu_367 and Ocu_371) were only recovered when the strain isolated in 2011 (Vib-oc-097) was not present, suggesting potential differences in phage infectivity that will be further discussed.

After three rounds of phage purification, a total of 10 viral isolates were obtained: 4 using the virome MVO1 as the source of phages with Vib-oc-097 as the host, 3 using the virome MVO2 with OcU_367 as the host, and 3 using MVO2 with OcU_371 as the host. The viral isolates were labeled with the name of the virome from which they were isolated, followed by the host strain and the plaque identifier number (Table 2).

**Table 2:** Genomic characteristic of the newly isolated *V. mediterranei* phages.

	Phage ID	*V. mediterranei* host	Virome used as phage source	Genome length (bb)	GC	Coding density	ORFs
*P. adelos*	MVO1_97.1	Vib-Oc-097	MVO1 (targeted virome with all Vm strains)	43 383	56.5	98.08	65
	MVO1_97.6			43 333	56.5	98.02	64
	MVO1_97.13^a^			43 350	56.5	98.08	65
	MVO1_97.16			43 269	56.5	98.21	65
*P. lipares*	MVO2_367.4	OcU_367	MVO2 (targeted virome with strains isolated in 2015)	44 317	57.8	97.47	63
	MVO1_367.8			44 198	57.8	97.48	63
	MVO1_367.14^a^			44 651	57.9	97.22	62
	MVO1_371.6	OcU_371		44 502	57.8	97.23	63
	MVO1_371.7			44 438	57.8	97.38	63
	MVO1_371.8			44 198	57.8	97.16	63

a Representative phages for each phage species.

### Genomic characterization of the phages infecting *V. mediterranei*

**Figure 3:**
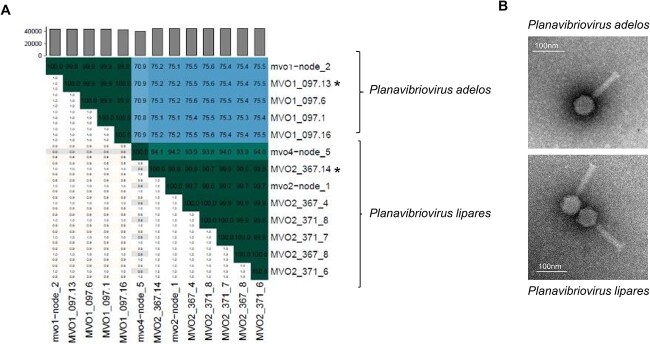
Characterization of *V. mediterranei* phages; (A**)** heatmap generated using VIRIDIC software to compare phage genomes and identify the closet contigs recovered from the viromes; in the right half, the numbers inside the map represent the nucleotide identity values for each genome pair, rounded to the first decimal; in the left half, the aligned genome length is indicated * indicates the representative phage for each species; (B) transmission electron microscopy images of *P. adelos* and *P. lipares* virions.

**Figure 4:**
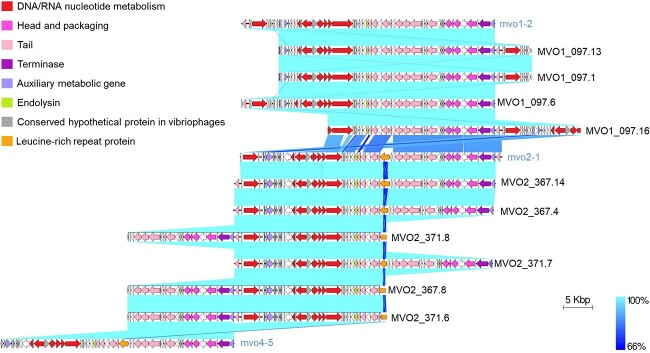
Easyfig homology diagram of isolated phages and the closest contigs recovered from viromes using BLASTn; functional gene groups are indicated in different colors (see the legend); the blue regions between the genome maps indicate the level of identity from 66% to 100% (see the legend on the right); genome labels with lowercase are from virome assemblies and labels in uppercase from isolated phages.

The isolated viruses infecting *V. mediterranei* had genome sizes of 43–45 kb, which matched the size observed by pulsed-field gel electrophoresis. The genomes had GC contents of 56%–58% and presented terminal redundancy (Table 2). They had no homology at the nucleotide level with previously isolated viruses in databases, including those infecting *Vibrio* spp. (see Supplementary Fig. 1 for a proteomic tree). Nucleotide-based intergenomic similarities calculated using VIRIDIC [36], which implements the traditional algorithm used by the International Committee on Taxonomy of Viruses (ICTV), confirmed that these isolated viruses constituted a new genus (>70% nucleotide similarity) and two species (>95% nucleotide similarity) (Fig. 3A). Transmission electron microscopy revealed that both species exhibited head–tail morphologies, indicating their classification within the Caudoviricetes class (Fig. 3B). We propose the name *Planavibriovirus* for the new genus. The genus name comes from the Latin name of the place of isolation (Tabarca Island, Planaria in Latin). The two species are named *P. adelos* (invisible in Greek) and *P. lipares* (persistent in Greek) respectively, in reference to their specific characteristic explained below (Fig. 4).

The first species (*P. adelos*, represented by the phage MVO1_097.13) includes the four phages isolated using the host strain Vib-oc-097 and the targeted virome MVO1. The second species (*P. lipares*, represented by the phage MVO2_367.14) contains phages isolated using the host strains OcU_371 and Ocu_367 and the targeted virome MVO2. All the genomes showed a high coding density (from 97.2% to 98.3% of the genome), and harbored genes coding for terminases, which is a hallmark of the Caudoviricetes class. Both viral species exhibited a consistent genome organization across three modules: virion morphogenesis (encompassing all the head and tail genes), DNA packaging and genome replication, and host cell lysis (Fig. 4). These two viral species also carried two distinct auxiliary AMGs: phosphoadenosine phosphosulfate reductase (PAPS) and FtsK/SpoIIIE-like protein (Fig. 4). Phosphoadenosine phosphosulfate reductase, previously observed in vibriophages [43, 44], has recently been recognized for its role in phage defense against the DNA phosphorothioation–based host defense system known as Dnd [43]. The Dnd defense system was detected in the Ocu_367 strain, but the vibriophages under study are able to infect both the Ocu_367 strain (which possesses the defense system) and the Ocu_371 strain (lacking the defense system). Hence, in this particular case, the PAPS gene does not seem to confer resistance against the Dnd system. Nevertheless, it has also been demonstrated that during infection, virus-encoded sulfur assimilatory enzymes like PAPS can help maintain a steady supply of this nutrient, enhancing energy production necessary for efficient phage replication [45]. Thus, the presence of these AMGs in the vibriophages suggests their ability to induce metabolic alterations during host infections and potentially play a significant role in biogeochemically relevant processes within the coral holobiont. The other AMG found, the FtsK/SpoIIIE-like protein, has been previously observed in bacteriophages and plasmids, and it likely serves an auxiliary role in facilitating phage DNA movement across the cell membrane [46].

The only distinction between the two viral species was an insertion of two open reading frames (ORFs) within the tail module of *P. lipares* (Fig. 4). One of these proteins, which showed the highest diversity among *P. lipares* genomes and also in the virome from which phages were isolated (Supplementary Fig. 2), exhibited homology to a leucine-rich repeat (LRR) protein (10% probability, hhpred). LRR proteins have been reported mainly in eukaryotes, bacteria, and viruses that predominantly infect eukaryotic hosts [47]. These proteins are crucial in the innate immune response of animals, including corals, recognizing specific molecular structures shared by pathogenic microorganisms like lipids, proteins, lipopolysaccharides (LPSs), lipoteichoic acid, and bacterial DNA [48]. To identify LRR proteins in the coral *O. patagonica*, we used a 69-Gbp metagenome from a colony collected in 2016 (unpublished results). Twenty LRR proteins could be identified, 16 of which were found to be related to LRR domains in other coral species through a blast against NCBI nr database (percentage of amino acid identity >50%). In these LRR proteins, a highly conserved segment (HCS) consisted of nine residues, with the consensus sequence LxxLxLxxN, was identified and this HCS was also detected in the vibriophage protein. Hence, the coral and the vibriophages isolated from its mucus layer share LRR-conserved domains. Recently, it has been proposed that LRR genes can be transferred horizontally between bacteriophages and their hosts [46]. Based on this, and aware of the obvious differences with our system, we hypothesize that these vibriophages could have acquired the LRR-conserved domains from the coral, allowing both corals and phages to utilize these proteins for recognizing bacterial pathogens or hosts, respectively, implying a significant role for LRR-mediated interactions among phages, bacteria, and corals.

During the assembly of the *P. adelos* phage (MVO1_097.13) a second contig with 36 782 bp was also assembled. Further investigation revealed that it corresponded to a prophage integrated into the *V. mediterranei* strain Vib-oc-097 genome, which was used as host for *P. adelos* replication (Supplementary Fig. 3). We propose the name *Mediterraneivibriovirus evadens* for this phage species. The genus name comes from the host name and the species means invisible in Greek. This virus showed a GC content of 43.5% and had no homology at the nucleotide level in databases with previously isolated viruses, including those infecting *Vibrio* spp. (see Supplementary Fig. 1 for a proteomic tree). In the genome, 56 coding sequences (CDS) were identified and consisted of head and tail genes, transcriptional regulation genes as the CI-like repressor, DNA packaging and genome replication, and host cell lysis as one endolysin, together with a terminase, which suggests that this phage belongs to *Caudoviricetes.* Host-pathogenicity-related genes were not identified in the lysogenic phage genome (Supplementary Fig. 4). Prophages are typically induced to become lytic under stressful environmental conditions or when host survival is threatened. Recent *in vitro* studies have revealed that quorum sensing molecules have the ability to induce prophages in pathogenic *V. cholerae*, depending on the availability of potential hosts beyond the infected cells [45]. Our findings provide evidence that the *V. mediterranei* prophage may become lytic when the lytic phage *P. adelos* infects its host. This induction could be promoted by an effect resulting from lysis such as the detection of a decrease in host density. The induction of prophages, triggered by lytic infections within the coral mucus layer, could potentially drive the subsequent lysogenic conversion of other *Vibrio* strains provided that the induced prophage is able to lysogenize them. This would be an ecologically relevant side effect of infection, still to be explored in a natural setting.

### Host range

To test the host specificity of *V. mediterranei* phages, we conducted an infection experiment using six additional *V. mediterranei* strains, as well as strains from the worldwide coral pathogen *V. coralliilyticus* (Supplementary Table 1). Our findings revealed that all the isolates belonging to *P. adelos* displayed a narrow host range, as they were only able to infect the Vib-oc-097 strain. In contrast, isolates classified as *P. lipares* exhibited a broader host range, infecting three distinct *V. mediterranei* strains, which were obtained simultaneously from the same location but from different *O. patagonica* colonies. Variations in host susceptibility to distinct *P. lipares* phage strains were observed (Table 2). As discussed above, the main difference among these strains lies in only one protein present in the tail module, which could be involved in phage–host interactions.

### *V. mediterranei* phage dynamics within *O. patagonica* mucus layer

**Figure 5:**
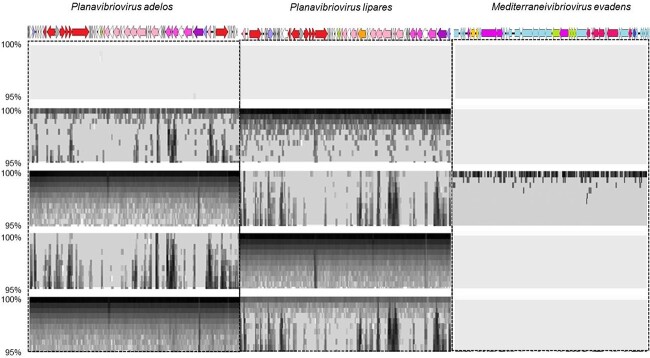
Fragment recruitment plot of virome reads against *V. mediterranei* lytic phages (*P. adelos* and *P. lipares*) and the prophage (*M. evadens*); the horizontal axis of each panel corresponds to one viral species from the indicated reference microbial genome; the vertical axis indicates the sequence identity of an alignment between a virome sequence read and the reference genomic sequence; identities range from 95% (bottom) to 100% (top).

A thorough analysis of the targeted viromes revealed the presence of three contigs (mov1-node2, mvo2-node1, and mvo4-node5) that displayed significant similarity (93.4%–99.9%) to the two isolated viral species (Fig. 3). This confirms that the genomes of the isolated vibriophages were also assembled from the targeted viromes, validating the consistency of our findings using both culture-based and metagenomic approaches. These viral genomes were not detected in the natural mucus sample, probably because their abundances were under the metagenomic detection limit (Fig. 5). However, these phages had 10- to 1000-fold higher abundances in the targeted metagenomes, which confirms the success of viral enrichment (Fig. 2).

The recruitment plot of sequence reads from the cultured vibriophages against the different viromes (Fig. 5) indicated that the abundances of these phages significantly increased in the presence of their hosts, representing over 95% of the reads in the targeted metagenomes. This increase was likely due to their active reproduction during incubation with their hosts. In turn, this allowed the successful isolation of the most abundant viruses generated during the enrichments. Furthermore, our results (Table 3) confirm the susceptibility of *Vibrio* strains isolated in 2011 and 2015 (8 and 4 years, respectively, before the experiment was conducted). The success of phages on bacterial hosts from the past could be attributed to changes in phage diversity over time, as this has been previously reported in other environments [49, 50]. This suggests that *Vibrio* strains isolated “in the past” (in this case, in 2011 and 2015) and preserved in the laboratory that were no longer exposed to phage selection could be more susceptible to phages from the environment that could overcome the resistance of these host strains by coevolution with the contemporary *Vibrio* strains.

**Table 3:** Results of cross-infection experiment using *V. mediterranei* strains as host, only positive results are shown. Each number indicates the number of PFUs per milliliter, reached after 24 h at 30°C of the phage (row names) was incubated with the host (column names).

Year	2011	2015		
Location	Spain	Spain		
Host	*O. patagonica*	*O. patagonica*		
	Vic-Oc-097	Ocu_367	Ocu_368	Ocu_371
MVO1_097.1	3.4 × 10^10^			
MVO1_097.6	6.3 × 10^9^			
MVO1_097.13	8.0 × 10^9^			
MVO1_097.16	8.2 × 10^9^			
MVO2_367.4		6.1 × 10^9^	3.2 × 10^7^	1.5 × 10^6^
MVO2_367.8		5.4 × 10^9^	9.8 × 10^8^	6.7 × 10^9^
MVO2_367.14		4.0 × 10^9^	5.8 × 10^9^	
MVO2_371.6		2.5 × 10^8^	7.3 × 10^9^	2.5 × 10^9^
MVO2_371.7		3.3 × 10^8^	2.9 × 10^8^	5.1 × 10^9^
MVO2_371.8		5.9 × 10^8^	3.4 × 10^8^	3.7 × 10^9^

The abundances of these two vibriophage species increased even when the mucus was incubated at 28°C without the addition of hosts, as was evident from the fragment recruitment plots shown in Fig. 5. This finding suggests that these phages were present in the SLM and, especially *P. lipares*, were capable of infecting co-occurring vibrios present in the coral mucus at the time of sampling.

Both species were detected through PCR amplification in two coral samples collected during the 2015 heatwave in Tabarca, from where the corals in the present study were taken 4 years later. The genome fragment amplified from the corals was sequenced and found to be identical to the reference sequence. Three samples from surrounding seawater were also check and were negative. These finding confirms the presence of these phages in *O. patagonica* colonies under heat stress, likely due to the proliferation of *V. mediterranei*. This not only supports the potential role of these phages in the coral defense against *Vibrio* pathogens but also indicates that mucus layer is the reservoir of these phages and the persistence of these (or closely related) vibriophages in the system.

These phages that were isolated from a specific location (Tabarca) were unable to infect strains isolated from other locations or from different invertebrates within the same location. This observation is consistent with other studies on phage isolations [24, 43, 51], which also indicated a preference of phages to infect hosts from the same site rather than hosts isolated from similar but geographically distant sites (Table 2).

The recruitment plot of the prophage integrated into the *V. mediterranei* strain Vib-oc-097 genome against the different viromes indicated the induction of this prophage in the targeted virome generated using the strain Vib-oc-097 (Fig. 5). This provides confirmation that the induction of this prophage could be promoted by the effect of lytic infection on its host.

### Concluding remarks

By leveraging a combination of cultivation and metagenomic approaches, we have discovered two phages able to infect the coral pathogen *V. mediterranei* and described key aspects on phage–*Vibrio* dynamic interactions in corals. These phages demonstrate a remarkable ability to proliferate rapidly when in the presence of their hosts, even though they are typically low-abundance specialists within the coral mucus layer. Additionally, our findings suggest that these phages show local adaptation to hosts, with a higher infectivity observed with hosts from the past rather than contemporary ones. Thus, the presence and dynamics of these phages in the coral mucus support the bacteriophage-adhering-to-mucus (BAM) model, which suggests that phages present in the coral mucus provide a nonhost-derived immunity to the coral [8]. The conserved LRR domain present in one vibriophage protein, which could be potentially involved in phage–host interactions and putatively acquired from the coral host, is also in agreement with the BAM model and may be pointing to a coevolution of phages and corals. This coevolution may be beneficial for both parties, with phage adherence benefiting the corals by limiting potentially pathogenic bacteria in the mucus layer and benefiting the phages by facilitating more frequent interactions with bacterial hosts.

Our research also points to the side effect of lytic infections in triggering prophage activation in *Vibrio* species. Under stressful conditions, such as a lytic infection of the host, prophages can excise themselves from the host genome, replicate, and complete the lytic cycle to infect new hosts. This process may facilitate the mobilization of genetic elements, including virulence factors, among vibrios in the coral mucus layer. Taken together, these findings highlight the intricate interplay among corals, vibrios, and phages and the potential importance of historical coral–*Vibrio*–phage interactions in maintaining coral health.

## Supplementary Material

Supplementary_Figure_1_wrae017

Supplementary_Figure_2_wrae017

Supplementary_Figure_3_wrae017

Supplementary_Figure_4_wrae017

Rubio-Portillo_et_al_2023_Supplementary_Table_1_wrae017

Rubio-Portillo_et_al_2023_Supplementary_Table_2_wrae017

Rubio-Portillo_et_al_2023_Supplementary_Table_3_wrae017

## Data Availability

The raw reads from viromes were deposited in the NCBI Sequence Read Archive under Bioproject accession number PRJNA1027786. Phage genomes sequences for each representative for each phage species are under accession numbers PP179306 (*Planavibriovirus adelos*) and PP179307 (*Planavibriovirus lipares)*.
