# Supplementary material for: Coral mucus as a reservoir of bacteriophages targeting Vibrio pathogens
Source: ISME J. 2024 Jan 31;18(1):wrae017. doi: 10.1093/ismejo/wrae017 (PMC10945359; doi:10.1093/ismejo/wrae017)
Supplement: Supplementary_Figure_1_wrae017 [file supplementary_figure_1_wrae017.pdf]

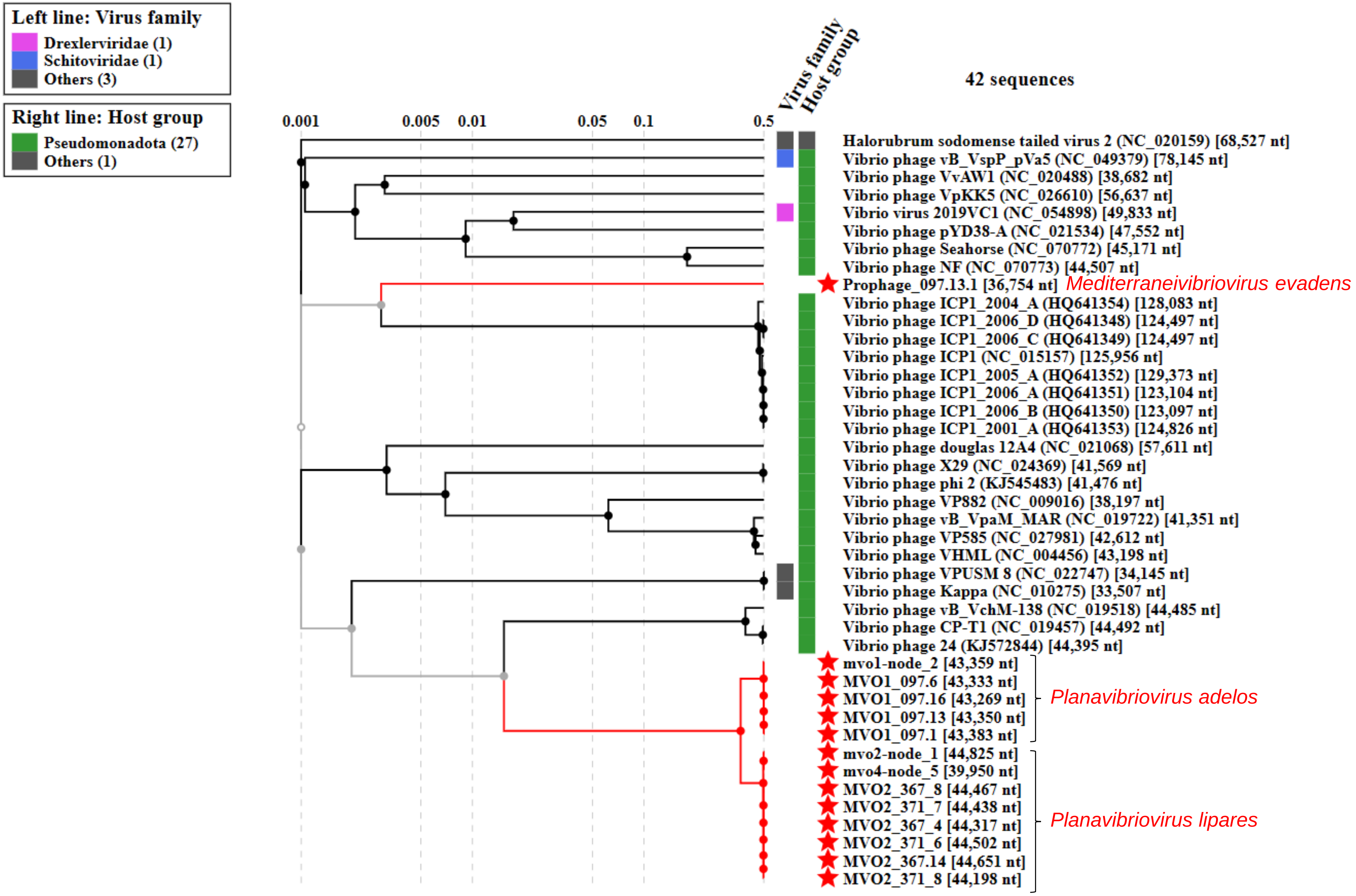
Left line: Virus family
Drexlerviridae (1)
Schitoviridae (1)
Others (3)
Right line: Host group
Pseudomonadota (27)
Others (1)
Virus family
Host group
42 sequences
0.001
0.005
0.01
0.05
0.1
0.5
Halorubrum sodomense tailed virus 2 (NC_020159) [68,527 nt]
Vibrio phage vB_VspP_pVa5 (NC_049379) [78,145 nt]
Vibrio phage VvAW1 (NC_020488) [38,682 nt]
Vibrio phage VpKK5 (NC_026610) [56,637 nt]
Vibrio virus 2019VC1 (NC_054898) [49,833 nt]
Vibrio phage pYD38-A (NC_021534) [47,552 nt]
Vibrio phage Seahorse (NC_070772) [45,171 nt]
Vibrio phage NF (NC_070773) [44,507 nt]
Prophage_097.13.1 [36,754 nt]
Mediterraneivibriovirus evadens
Vibrio phage ICP1_2004_A (HQ641354) [128,083 nt]
Vibrio phage ICP1_2006_D (HQ641348) [124,497 nt]
Vibrio phage ICP1_2006_C (HQ641349) [124,497 nt]
Vibrio phage ICP1 (NC_015157) [125,956 nt]
Vibrio phage ICP1_2005_A (HQ641352) [129,373 nt]
Vibrio phage ICP1_2006_A (HQ641351) [123,104 nt]
Vibrio phage ICP1_2006_B (HQ641350) [123,097 nt]
Vibrio phage ICP1_2001_A (HQ641353) [124,826 nt]
Vibrio phage douglas 12A4 (NC_021068) [57,611 nt]
Vibrio phage X29 (NC_024369) [41,569 nt]
Vibrio phage phi 2 (KJ545483) [41,476 nt]
Vibrio phage VP882 (NC_009016) [38,197 nt]
Vibrio phage vB_VpaM_MAR (NC_019722) [41,351 nt]
Vibrio phage VP585 (NC_027981) [42,612 nt]
Vibrio phage VHML (NC_004456) [43,198 nt]
Vibrio phage VPUSM 8 (NC_022747) [34,145 nt]
Vibrio phage Kappa (NC_010275) [33,507 nt]
Vibrio phage vB_VchM-138 (NC_019518) [44,485 nt]
Vibrio phage CP-T1 (NC_019457) [44,492 nt]
Vibrio phage 24 (KJ572844) [44,395 nt]
mvo1-node_2 [43,359 nt]
MVO1_097.6 [43,333 nt]
MVO1_097.16 [43,269 nt]
MVO1_097.13 [43,350 nt]
MVO1_097.1 [43,383 nt]
Planavibriovirus adelos
mvo2-node_1 [44,825 nt]
mvo4-node_5 [39,950 nt]
MVO2_367_8 [44,467 nt]
MVO2_371_7 [44,438 nt]
MVO2_367_4 [44,317 nt]
MVO2_371_6 [44,502 nt]
MVO2_367.14 [44,651 nt]
MVO2_371_8 [44,198 nt]
Planavibriovirus lipares
