# Supplementary figures and images for: Coral mucus as a reservoir of bacteriophages targeting Vibrio pathogens
Source: ISME J. 2024 Jan 31;18(1):wrae017. doi: 10.1093/ismejo/wrae017 (PMC10945359; doi:10.1093/ismejo/wrae017)

Hypothetical  
protein

Protein with leucine rich protein  
conservade domain

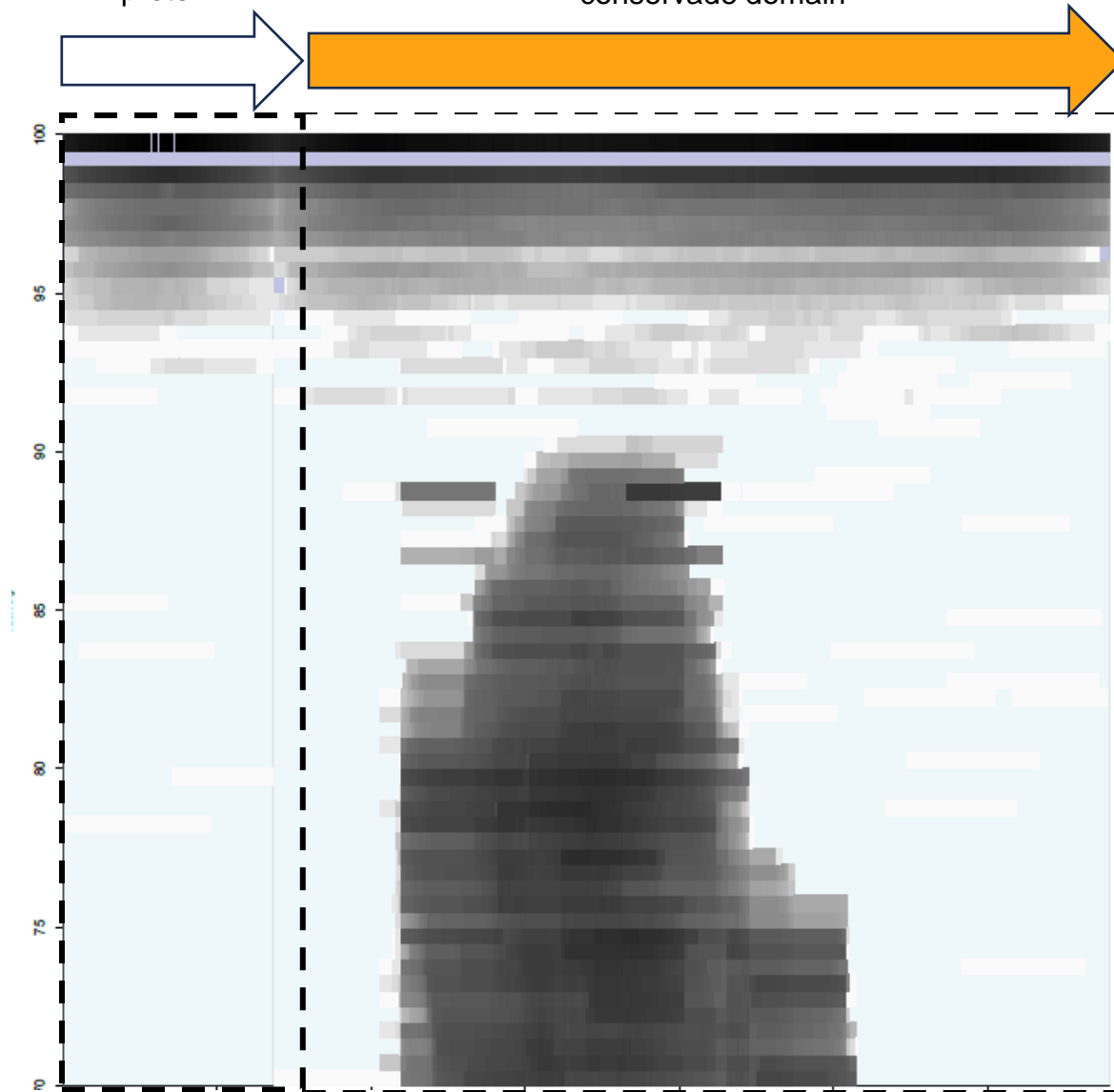

Supplement: Supplementary_Figure_2_wrae017 [file supplementary_figure_2_wrae017.pdf]

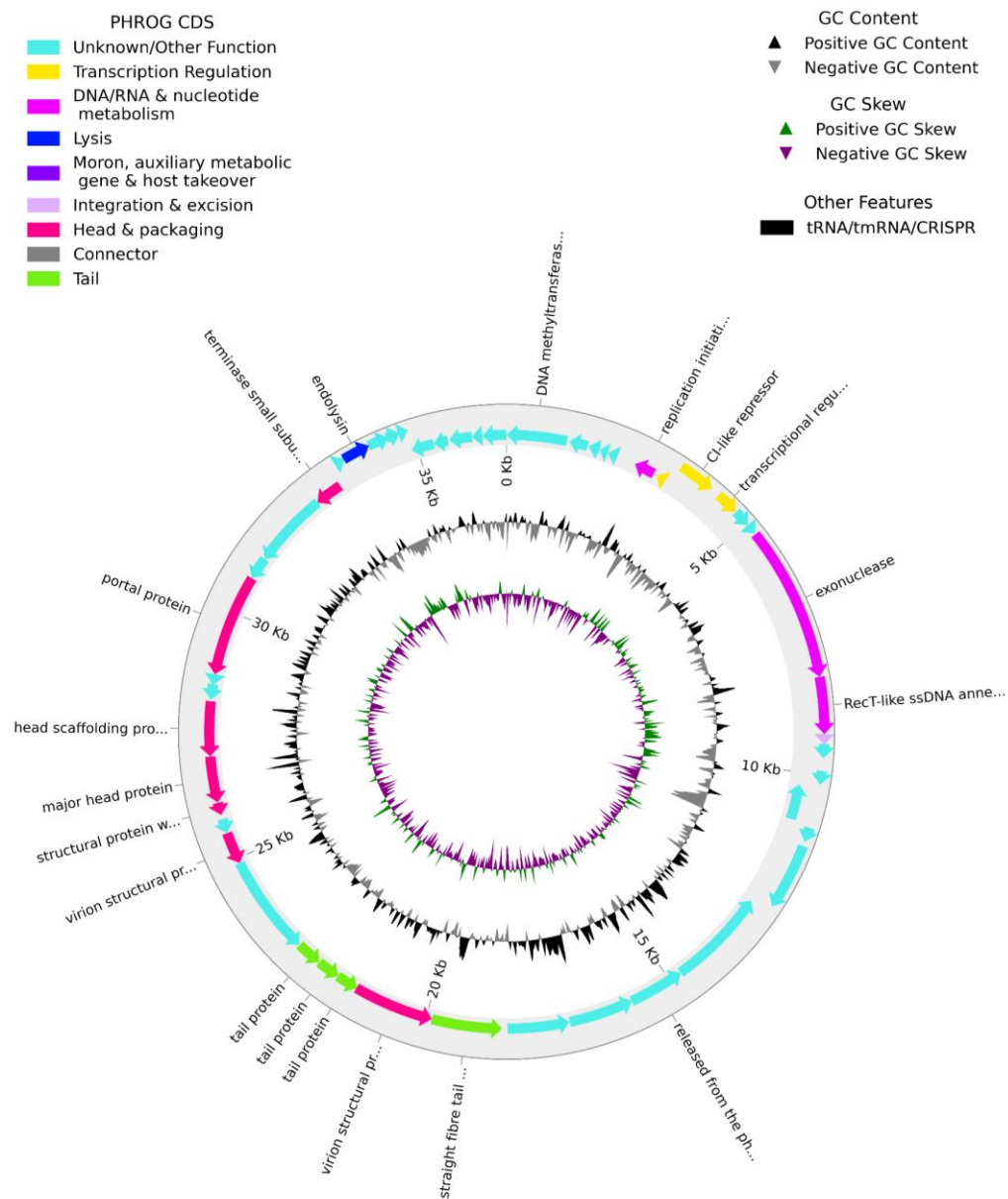

Supplement: Supplementary_Figure_4_wrae017 [file supplementary_figure_4_wrae017.pdf]
