# Supplementary material for: Coral mucus as a reservoir of bacteriophages targeting Vibrio pathogens
Source: ISME J. 2024 Jan 31;18(1):wrae017. doi: 10.1093/ismejo/wrae017 (PMC10945359; doi:10.1093/ismejo/wrae017)
Supplement: Supplementary_Figure_3_wrae017 [file supplementary_figure_3_wrae017.pdf]

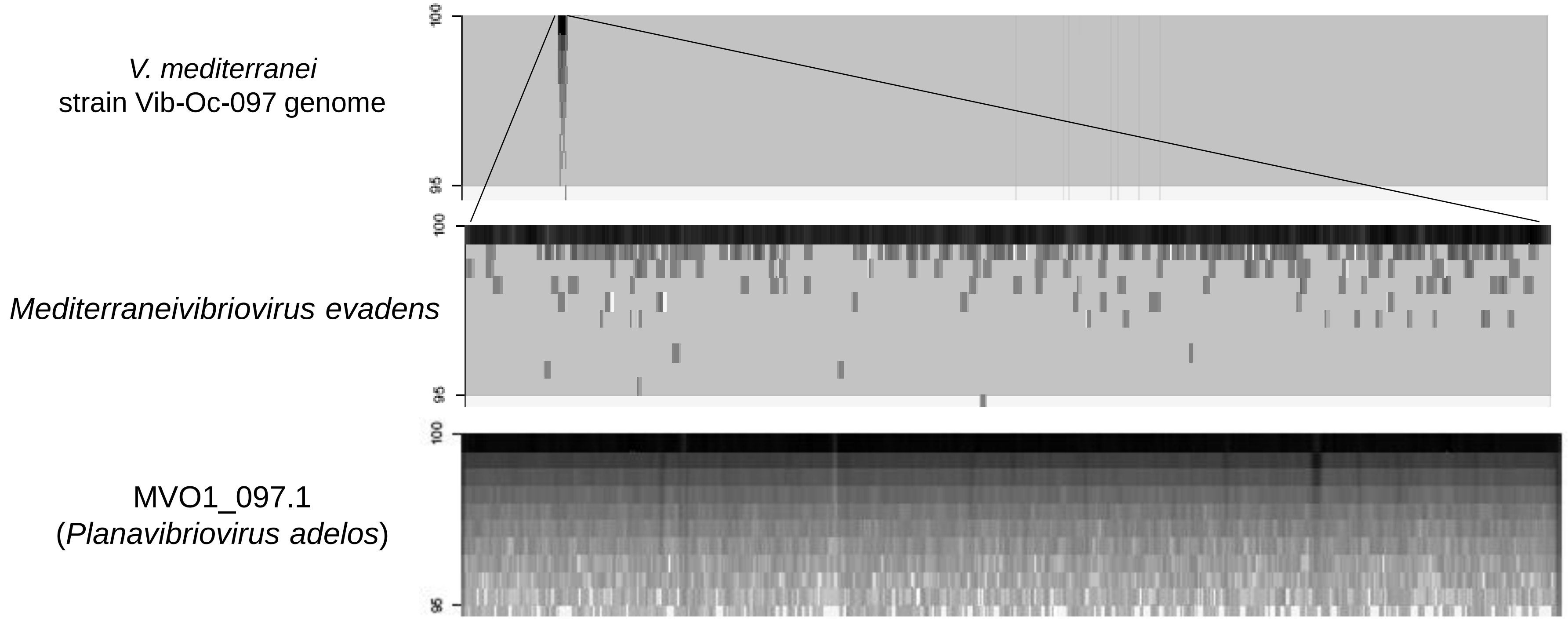
V. mediterranei
strain Vib-Oc-097 genome
Mediterraneivibriovirus evadens
MVO1_097.1
(*Planavibriovirus adelos*)
100
95
100
95
100
95
