# Supplementary material for: Coral mucus as a reservoir of bacteriophages targeting Vibrio pathogens
Source: ISME J. 2024 Jan 31;18(1):wrae017. doi: 10.1093/ismejo/wrae017 (PMC10945359; doi:10.1093/ismejo/wrae017)
Supplement: Rubio-Portillo_et_al_2023_Supplementary_Table_1_wrae017 [file rubio-portillo_et_al_2023_supplementary_table_1_wrae017.docx]

Supplementary Table 1. Strains used for cross infection experiment with the *Vibrio mediterranei* isolated phages.

| Vibrio strain | Year | Location | Host | Id |
| --- | --- | --- | --- | --- |
|  | 1981 | Spain (Valencia) | *Seawater* | CECT 623 |
| *Vibrio mediterranei* | 1995 | Israel | *Oculina patagonica* | CECT 7873 (AK-1) |
|  | 2011 | Spain | *Oculina patagonica* | Vic-Oc-096 |
|  |  |  |  | Vic-Oc-097 (CECT 30098) |
|  | 2012 | Spain | *Oculina patagonica* | Voc_213 |
|  |  |  | *Cladocra caespitosa* | Vc_0163 |
|  |  | Italy | *Oculina patagonica* | Voc_054 |
|  |  |  | *Cladocra caespitosa* | Vcl_029 |
|  | 2015 | Spain | *Oculina patagonica* | OcU_367 |
|  |  |  |  | OcU_368 |
|  |  |  |  | OcU_371 |
|  |  |  | *Sarcotragus fasciculatus* | IrU_303 |
|  |  |  |  | IrU_323 |
| *Vibrio coralliilyticus* | 2011 | Spain | *Oculina patagonica* | Vic-Oc-027 |
|  | 2012 | Spain | *Oculina patagonica* | Voc-156 |
|  |  |  | *Cladocra caespitosa* | Vcl-242 |
|  |  | Italy | *Oculina patagonica* | Voc-070 |
|  |  |  | *Cladocra caespitosa* | Vcl-151 |
|  | 2022 | Zanzibar | *Pocillopora damicornis* | LMG20984 |
