# Supplementary material for: Coral mucus as a reservoir of bacteriophages targeting Vibrio pathogens
Source: ISME J. 2024 Jan 31;18(1):wrae017. doi: 10.1093/ismejo/wrae017 (PMC10945359; doi:10.1093/ismejo/wrae017)
Supplement: Rubio-Portillo_et_al_2023_Supplementary_Table_2_wrae017 [file rubio-portillo_et_al_2023_supplementary_table_2_wrae017.docx]

Supplementary Table 2. Overview of iPHop host predictions for high-quality viral OTUs.

| **Contig** | **IPhoP results** |
| --- | --- |
| mvo1-node-1 | d__Bacteria;p__Proteobacteria;c__Gammaproteobacteria;o__Enterobacterales;f__Vibrionaceae;g__Vibrio;s__Vibrio |
| mvo3-node-1 | d__Bacteria;p__Proteobacteria;c__Gammaproteobacteria;o__Enterobacterales;f__Vibrionaceae;g__Vibrio;s__Vibrio |
| mvo3-node-2 | d__Bacteria;p__Proteobacteria;c__Gammaproteobacteria;o__Pseudomonadales;f__Halieaceae;g__Luminiphilus;s__Luminiphilus |
| mvo4-node-1 | d__Bacteria;p__Proteobacteria;c__Gammaproteobacteria;o__Enterobacterales;f__Vibrionaceae;g__Vibrio;s__Vibrio |
| mvo4-node-12 | d__Bacteria;p__Proteobacteria;c__Gammaproteobacteria;o__Enterobacterales;f__Vibrionaceae;g__Vibrio;s__ |
| mvo4-node-3 | d__Bacteria;p__Proteobacteria;c__Gammaproteobacteria;o__Burkholderiales;f__Burkholderiaceae;g__Acidovorax |
| mvo4-node-6 | d__Bacteria;p__Proteobacteria;c__Gammaproteobacteria;o__Enterobacterales;f__Vibrionaceae;g__Vibrio;s__Vibrio |
| mvo4-node-8 | d__Bacteria;p__Proteobacteria;c__Gammaproteobacteria;o__Enterobacterales;f__Vibrionaceae;g__Vibrio;s__Vibrio |
| mvo4-node-9 | d__Bacteria;p__Proteobacteria;c__Gammaproteobacteria;o__Enterobacterales;f__Vibrionaceae;g__Vibrio;s__Vibrio |
| mvo5-node-103 | d__Bacteria;p__Proteobacteria;c__Gammaproteobacteria;o__Enterobacterales;f__Alteromonadaceae;g__Pseudoalteromonas;s__Pseudoalteromonas |
| mvo5-node-235 | d__Bacteria;p__Proteobacteria;c__Alphaproteobacteria;o__Sphingomonadales;f__Sphingomonadaceae;g__Sphingobium;s__Sphingobium |
| mvo5-node-25 | d__Bacteria;p__Proteobacteria;c__Alphaproteobacteria;o__Rhizobiales;f__Stappiaceae;g__Roseibium;s__ |
| mvo5-node-34 | d__Bacteria;p__Cyanobacteria;c__Cyanobacteriia;o__Phormidesmiales;f__Phormidesmiaceae;g__PCC-7375;s__PCC-7375 |
| mvo5-node-46 | d__Bacteria;p__Bacteroidota;c__Bacteroidia;o__Flavobacteriales;f__Flavobacteriaceae |
| mvo5-node-61 | d__Bacteria;p__Bacteroidota;c__Bacteroidia;o__Sphingobacteriales;f__Sphingobacteriaceae;g__Sphingobacterium;s__ |
| mvo5-node-63 | d__Bacteria;p__Proteobacteria;c__Alphaproteobacteria;o__Rhizobiales;f__Rhizobiaceae;g__Rhizobium;s__Rhizobium |
| mvo5-node-69 | d__Bacteria;p__Proteobacteria;c__Gammaproteobacteria;o__Pseudomonadales;f__Halieaceae;g__Luminiphilus;s__ |
| mvo5-node-72 | d__Bacteria;p__Proteobacteria;c__Gammaproteobacteria;o__Enterobacterales;f__Enterobacteriaceae |
| mvo5-node-80 | d__Bacteria;p__Bacteroidota;c__Bacteroidia;o__Flavobacteriales;f__Flavobacteriaceae |
