# Supplementary material for: Coral mucus as a reservoir of bacteriophages targeting Vibrio pathogens
Source: ISME J. 2024 Jan 31;18(1):wrae017. doi: 10.1093/ismejo/wrae017 (PMC10945359; doi:10.1093/ismejo/wrae017)
Supplement: Rubio-Portillo_et_al_2023_Supplementary_Table_3_wrae017 [file rubio-portillo_et_al_2023_supplementary_table_3_wrae017.docx]

Supplementary Table 3. Results of spot-test using the targeted metaviromes and the three *Vibrio mediterranei* hosts.

|  | MVO1  (Targeted metaviome using all Vm strains) | MVO2  (Targeted metaviome using strains OcU_367 and OcU_371) | MVO3  (Targeted metaviome using strain Vic-Oc-097 | MVO4  (Coral mucus at 28ºC) | MVO5  (Coral mucus) |
| --- | --- | --- | --- | --- | --- |
| Vic-Oc-097 | + | - | + | - | - |
| OcU_367 | - | + | - | - | - |
| OcU_371 | - | + | - | - | - |
